# Assessing the feasibility of police initiation of HIV post-exposure prophylaxis for sexual violence survivors in Lusaka, Zambia

**DOI:** 10.1186/1753-6561-9-S4-A3

**Published:** 2015-07-07

**Authors:** Mary T Zama, Mardieh Dennis, Jessica Price, Stephanie M Topp

**Affiliations:** 1 Population Council, Private Bag 319X, Ridgeway, Lusaka, Zambia; 2 Centre for Infectious Disease Research in Zambia, PO Box 34681, Lusaka, Zambia; 3 Nossal Institute for Global Health, University of Melbourne, Level 4, 161 Barry Street, Alan Gilbert Building, Carlton 3010, VIC, Australia

## Background

Globally, more than 1 in 3 women have experienced physical or sexual violence (SV) from intimate partners or SV from non-partners [1]. Furthermore, over 10% of all girls are estimated to have experienced a forced sexual act, with the highest rates of SV against girls found in sub-Saharan Africa [2].

Although public recognition of SV is growing in Zambia, reliable data on the nature and extent of such violence is limited. Approximately 20% of Zambian women aged 15 to 49 have experienced some form of sexual violence; however, this is likely underestimated due to underreporting [3]. Previous research in Zambia suggests that exposure to SV is equally pervasive among adolescent girls [4,5].

The risks associated with SV, especially among young women, are numerous. Immediate health consequences include unwanted pregnancy, physical trauma, mental distress and acquisition of HIV and other sexually-transmitted infections. The linkage between sexual and gender-based violence (SGBV) and risk of HIV has been well documented in Africa and is especially pronounced in countries with high HIV prevalence, such as Zambia [3,6-8].

Growing awareness of these negative consequences of SGBV led the Government of Zambia to develop a set of national guidelines for the management of SGBV, highlighting the need for a response system linking the health, police, and social services sectors. A critical component of this response is the prevention of HIV infection in SV survivors through the provision of preventive anti-retroviral therapy, or HIV post-exposure prophylaxis (PEP). The initial dose of PEP must be taken within 72 hours of exposure to HIV [9].

Given the time sensitivity of PEP and the fact that police and health services are often the first points of contact for SV survivors, strong coordination between these two sectors is central to the effective medical management of SV cases in Zambia [10]. Building on the results of previous research in Zambia, which demonstrated that trained Victim Support Unit (VSU) police officers could effectively administer the emergency contraception pill to SV survivors, the Population Council, Zambia Police Service, and Ministry of Health aimed to assess the feasibility of having trained VSU police officers safely and effectively provide a PEP starter dose to SV survivors with immediate referral to comprehensive medical services [10].

## Materials and methods

### Study sites

The feasibility study was conducted at two police stations and four associated police posts in two high-density, low-income communities in Lusaka. The combined catchment area population of the two communities is approximately 296,000. These sites were purposively selected to represent urban areas with high SV prevalence rates.

### Intervention

The intervention involved two complementary components: (i) multi-sectoral training sessions to create an enabling environment for effective SV case management and (ii) VSU police officer provision of a 3-day PEP starter dose to SV survivors.

Service providers and policy makers in the police, medical, and social services sectors were trained on what constitutes SGBV; risks associated with SGBV; the rights of SV survivors; the Zambian government's multi-sectoral approach to managing SV cases; and specific interventions available to SV survivors, including PEP.

VSU police officers and medical personnel also received additional training on how to safely and effectively initiate SV survivors on PEP at the police station with immediate referral to the nearest tertiary hospital for forensic evidence collection and comprehensive medical assessment and treatment. A screening checklist was developed to guide VSU officers in assessing PEP eligibility. Individuals were considered eligible for police-initiated PEP if they met the following criteria:

i. Reported an SV incident involving penetrative sex

ii. Presented to the VSU within 72 hours of the incident

iii. Age ≥10 years

iv. Not currently on antiretroviral therapy (ART)

Children aged less than 10 years were excluded due to the complexities of calculating ART dosages based on weight for this age group. These children were referred directly to the nearest One-Stop Centre, where they could receive child-focused medical, psychosocial, and police services.

### Data collection and analysis

Each police station and post was provided with a survivor log book where information on client demographics and the date, time, and type of SV incident was recorded. Monthly monitoring visits were conducted at all participating police stations and posts to review the survivor screening checklists for cases reported during the previous month and to transcribe service data from the survivor log books. Descriptive statistical analysis of the service data was performed using Stata 13 software.

## Results

### Case reports

A total of 207 SV cases were reported at 2 police stations and 4 police posts during the project period. All 207 cases were female. Of note, 85% of cases involved girls under the age of 16 years, with the mean age of report being 13 years.

### Referrals

The intervention protocol mandated that all SV cases be referred to the nearest tertiary hospital for further testing and treatment. According to the survivor log book records, 96% (n = 199) of survivors who reported to the VSU were given the appropriate medical report form for referral to the hospital. However, only 2% (n = 4) were accompanied to the hospital by a police officer, as stipulated by the national SGBV case management guidelines. Since so few cases were accompanied to the hospital, the proportion of cases that actually received additional medical services is unclear.

### PEP delivery

**Figure 1:**
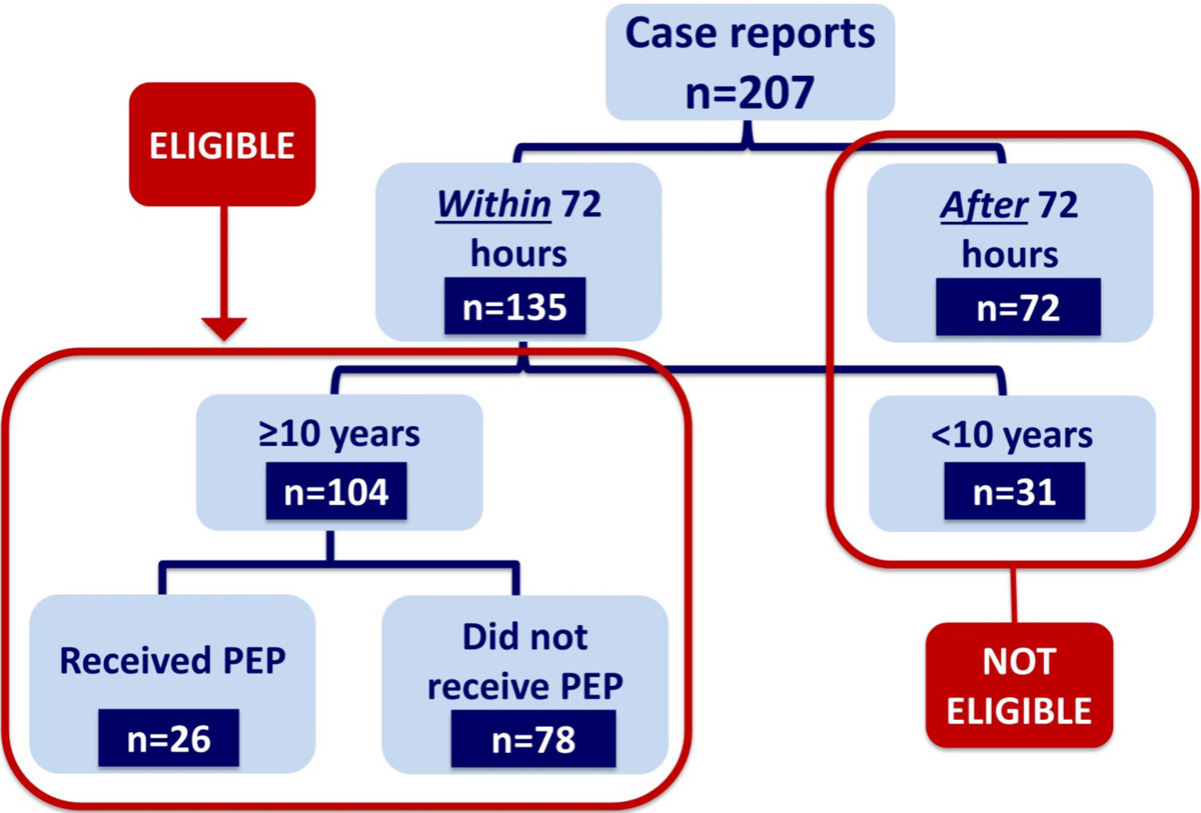
**Police delivery of PEP (November 2012 - October 2013)**.

Approximately 50% (n = 104) of the 207 case reports were eligible to receive PEP (Figure 1). Only 25% (n = 26) of eligible survivors were initiated on PEP by the police. Notably, less than half (n = 49) of all eligible cases reported at police stations or posts during the VSU officers' official working hours. While 33% of eligible survivors who reported during official working hours received PEP, only 18% of those who reported on nights or weekends received PEP. No adverse events were reported as a result of trained VSU police officers providing PEP during the study period.

## Conclusions

The results from this feasibility study demonstrate that police officers can safely and effectively provide SV survivors with a 3-day starter pack of PEP and refer them to health services for follow-up. However, the study also highlights challenges that inhibit greater access to police-initiated PEP. There is a need to restructure VSUs to ensure that they are open 24 hours per day and adequately staffed. Training in SV and the provision of PEP could be extended to non-VSU officers to enhance staffing. Given the profile of SV survivors seeking services at police stations and posts in Zambia (with the vast majority being aged 16 and below), police and health services must also be tailored to meet the unique needs of child survivors of SV. Additional funding has since been obtained to begin to explore how to address this issue in police stations in Zambia.
